# Supplementary material for: Are carbon emissions trading and green financial instruments synergistic? -Comprehensive quantitative research based on content analysis
Source: PLoS One. 2024 Mar 7;19(3):e0298601. doi: 10.1371/journal.pone.0298601 (PMC10919601; doi:10.1371/journal.pone.0298601)
Supplement: S1 Data — (ZIP) [file pone.0298601.s001.zip › Data/地级市Z_F工作报告.zip!/╣π╬≈/║╙│╪╩╨/└┤╫╘╣1⁄2╓┌║┼í╛┬φ┐╦╩2╛▌═°í┐_2015.docx]

各位代表：

现在，我代表市人民政府向大会作政府工作报告，请予审议，并请政协委员和列席会议的同志提出意见。

一、2014年工作回顾

2014年，面对错综复杂的形势，市人民政府在自治区党委、政府和市委的坚强领导下，着力强化重点治贫促发展、重力治乱保民安、重心治政树新风的“三治”理念，以“稳进突围、提质发展”为总基调，以开展党的群众路线教育实践活动和深化产业增量提质、民生保障提升、开发扶贫攻坚、城乡新貌新风、干部执行力提升“五大工程”为抓手，奋力推动工业发展、农业发展、交通基础设施建设、巴马长寿养生国际旅游区起步建设、城乡建设和干部作风转变“六大突围”，全市呈现经济稳中向好、民生持续改善、社会和谐稳定的良好局面。初步统计，全市地区生产总值601亿元，增长8.1%；财政收入54.67亿元，增长8.84%（不扣除不可预见的政策性退税1.98亿元，完成56.65亿元，增长12.8%）；全社会固定资产投资401.6亿元，增长15%；规模以上工业总产值374.33亿元、增加值137.26亿元，分别增长10.8%、14.5%；社会消费品零售总额223.79亿元，增长12.5%；城镇居民人均可支配收入21520元，增长9.5%；农村居民人均纯收入5770元，增长11%；招商引资内资到位249.23亿元，增长8.3%，外资到位1.01亿美元，增长44.3%，增幅排全区第一；居民消费价格涨幅控制在2.8%。

“十二五”规划45项经济社会发展指标中，经过4年来的努力，已有农村减贫人口、九年义务教育巩固率、主要劳动年龄人口平均受教育年限、科技进步贡献率、森林覆盖率、旅游总收入等29项提前完成，占指标总数的64.5%。

一年来，我们主要做了以下工作：

（一）主动适应新常态，经济保持平稳健康发展。年初经济下行压力大，有色金属、蔗糖、化工、纺织等传统产业市场持续低迷，一季度主要经济指标均是负增长。我们多次召开政府常务会议、专题会议，研究产业、项目、园区、巴马国际旅游区建设等问题，提出稳增长的37条具体措施，出台支持中小企业发展助保金贷款办法、促进糖业可持续发展意见、培育“四上”企业办法、非公有制强优企业发展奖励办法等措施，努力寻求经济发展新常态下促进发展的新办法、新动力和新增长点，扭转了经济下滑局面，呈现“筑底蓄势、稳健上行”态势。

（二）调结构促转型，三次产业协调发展。第一产业稳中有进，第二产业转型成效明显，第三产业比重有所提高，产业结构出现积极变化。

工业转型升级取得新进展。工业园区培育壮大，全市11个工业园区（集中区）完成总产值215亿元，增长10.7%，完成增加值57亿元，增长10%，园区基础设施建设完成投资6.3亿元，增长21%，其中，大任产业园被列入自治区重点园区，累计征地13661亩，入园二级公路全线贯通，其他基础设施和首批出城入园企业建设加快推进。电力产业总产值首次突破100亿元，成为我市第三个百亿元产业。完成年产新型干法水泥1200万吨项目布局，产能跃居全区前列。有色金属产业集约化、规模化程度明显提升，生态环保型有色金属产业示范基地建设取得积极成效，有色金属产品产量较快增长，增加值增长6.8%。规模以上有色金属、电力、食品加工、纺织、化工等五大支柱产业完成工业总产值293亿元，增长14%。新兴产业加快发展，酿酒、优质饮用水产业产能、产值稳居全区首位。泓驰公司新能源低速电动汽车项目投产，鑫锋公司新型蓄电池项目、恒业公司丝绸印染项目加快推进，低速电动汽车、新型蓄电池、丝绸印染等产业抢占发展先机。万元地区生产总值能耗0.78吨标准煤，下降7%，单位工业增加值能耗下降10.87%，超额完成年度节能任务。颁发首届“市长质量奖”，巴马丽琅成功创建中国驰名商标，新增15件广西著名商标、8个广西名牌产品。实施科技创新项目10个，通过自治区级科技成果鉴定3项。

农业稳中提质。全市粮食总产量106.52万吨，创8年来新高。桑蚕、糖料蔗、食用菌、优质水果、养殖等特色优势农业规模化、基地化、集约化、标准化程度提升。桑园面积78.59万亩，鲜茧产量10.77万吨，继续稳居全区地级市第一。牛出栏19.75万头，增长2.12%，山羊出栏81.85万只，增长3.28%，内陆大水面开发面积21.29万亩，水产品产量7.33万吨，增长6.04%，牛、山羊饲养量和出栏量及内陆大水面开发面积继续保持全区第一，新增农业部“水产健康养殖场”3个、“畜禽渔业标准化养殖场”9个。“三品一标”认定面积43.38万亩，居全区前列。农民合作社和家庭农场分别新增548家、117家，达1122家、210家。新认定市级农业产业化龙头企业8家、达73家。建立林业专业合作社111个，全市林业总产值147亿元，林下经济产值35亿元。开发了一批富硒农产品，打响长寿之乡富硒农产品品牌。农作物耕种收综合机械化率提高3个百分点，达39%。新增恢复、改善灌溉面积17.6万亩。

现代服务业加快发展。启动实施巴马长寿养生国际旅游区基础设施建设大会战，完成投资17亿元。组建河池巴马国际养生旅游学校。环江喀斯特成功入选世界自然遗产名录。天峨龙滩大峡谷国家森林公园晋级4A级景区。罗城地质公园、都安地下河地质公园、都安澄江湿地公园、东兰坡豪湖湿地公园晋级国家级公园。宜州市荣获“中国长寿之乡”称号。金城江姆洛甲景区和环江牛角寨景区被评为广西生态旅游示范区。制定加快旅游业发展扶持奖励办法，出台金融支持旅游业发展的实施意见，获得自治区扶持旅游发展专项资金7760万元，广西农村信用联社授信2亿元信贷资金支持旅游发展。全市接待国内外游客1539万人次，增长19%；旅游总收入147亿元，增长30%，其中，巴马长寿养生国际旅游区接待国内外游客851万人次，增长21%；旅游总收入81亿元，增长30%。市中心城区11个专业市场全部投入使用，市场体系进一步完善。金融保险保障能力提高，新增存款84.3亿元，增长11.55%，新增贷款51.99亿元，增长12.39%，近七成投向“三农”和小微企业。分别与中国人保财险广西分公司、国海证券公司签订战略合作协议，进一步扩大政策性农业保险覆盖面，5家企业在北部湾股权交易所挂牌。新成立3家小额贷款公司，总数达13家。

（三）大力抓好开发扶贫，16.5万群众实现脱贫。开发扶贫模式不断创新。新增4个广西“十百千”产业化扶贫示范工程项目，成为全区唯一实现项目全覆盖的地级市。“整乡扶贫”示范、水利整乡（村）推进在全区率先铺开。生态移民搬迁模式在全区推广，移民6279户26498人，搬迁规模和资金总量排全区第一。探索金融扶贫新模式，创建黔桂“四地”金融扶贫连片试验区。在全区率先完成684个贫困村，100.248万贫困人口精准识别、建档立卡工作。推广环江大安“整乡推进”扶贫模式，11个县（市、区）各抓1个乡镇作为“整乡推进”建设示范点，实施配套建设项目962个，累计投入资金9.34亿元，各示范点面貌明显改善。大力发展核桃、桑蚕等十大扶贫主导产业，新增核桃种植95.9万亩，全市核桃总面积达172.5万亩，核桃苗木实现本地化供应。整合中央小型农田水利重点县和土地出让收益金2.8亿元，每个县（市、区）实施1个“五小”水利整乡（村）推进建设示范。创新人口计生扶贫帮扶模式，实施计生小额贴息贷款帮扶项目，13万户贫困计生家庭受益。建设通村水泥路168条1468公里，屯级道路898条1053公里，项目全部竣工后建制村公路通畅率将达90%、自然屯（20户以上）道路通畅率将达77%以上。岩滩水电站水库移民遗留问题处理规划强力实施，移民口粮补助标准得到提高，移民子女教育扶持政策启动实施。在全国首个“扶贫日”启动河池市“千企助百村”行动，首批34家结对帮扶非公企业捐赠资金、物资821万元，扶贫产业项目意向投资19.5亿元，各县（市、区）非公企业积极响应，捐款捐物2172万元。开展“两广”对口帮扶职业教育协作试点，帮扶协作领域进一步拓宽。市扶贫办荣获“全国社会扶贫先进集体”荣誉称号。

（四）加快重大项目建设，发展后劲不断增强。深化市领导联系重大项目制度，出台政府投资项目管理办法、并联审批实施方案、容缺预审操作规程，以耕地占补平衡、低丘缓坡试点等措施，确保重大项目建设用地需求，推动一批重大项目实现开竣工。按照“抓好铁（路）公（路）机（场）、打通金水道”的思路，推动交通基础设施建设取得历史性突破，河池机场正式通航；河池至都安高速公路建成通车，西南出海大通道实现全线高速，开工建设河池至百色高速公路；环江华山至金城江、都安至武鸣（河池段）等二级公路基本贯通；河池港东兰港弄堂作业区试运行成功，西南水运出海中线通道龙滩水电站以下红水河航线全线贯通；贵阳经河池至南宁客运专线、黔桂铁路增建二线项目前期工作加快推进，罗城火车站建成使用。全年统筹推进重大项目248项，完成投资148.76亿元。岩滩水电站扩建工程，南丹懂托、拉纳水电站投产发电，新增发电装机容量66万千瓦。广维公司糖蜜制酒精项目一期、国投鱼峰水泥公司技改项目等一批重大产业项目竣工投产。县县通天然气工程加快推进，河池天然气末站具备供气条件，4个县（市）城区管网工程开工建设。一批重大水利项目加快建设，水库除险加固实施61座、完工55座，进度排名全区第一，建设农村饮水安全工程1486处，解决34.5万农村人口饮水不安全问题。

（五）抢抓机遇创优势，改革开放迈出新步伐。2014年是全面深化改革元年，我们紧紧围绕改革创新主线谋划、部署和推进各项工作。出台优化环境促进非公有制经济跨越发展的实施意见，全面落实工商注册登记制度改革、小微企业帮扶政策，激发大众创业、万众创新的活力，3月份改革以来，全市新登记企业3366户，注册资本78.2亿元，比上年同期增长2.5倍。建立不动产局际联席会议制度，农村宅基地和集体建设用地确权登记发证工作加快推进，累计发证49.1万宗。启动11个乡（镇）土地确权登记颁证试点。累计完成206家国有企业改革改制。推动国有资产集中统一监管，多种形式盘活国有资产，市直11家国有企业整体划入市国投公司。成立市矿业投资开发有限责任公司，建立矿产资源管控整合平台。食品药品监管体制改革走在全区前列，乡镇（街道）全部设立食品药品监督管理所，所有行政村落实协管员。

积极扩大对外交往合作，与国际友城泰国罗勇市实现首次互访，与澳大利亚洛根市签订建立友好城市框架协议，与世界杰出华商协会签订投资合作协议，引进碧桂园集团等一批知名大企业落户河池。与长三角投资集团共同举办PPP项目投资促进会，68家知名企业组建“中国PPP董事长联盟”。第十一届中国—东盟博览会签约37个项目，总投资达334.18亿元，签约金额再创新高。

（六）建设美丽河池，城乡面貌明显改观。实施了一批城镇建设重点项目，新型城镇化步伐加快，城镇化率达31.36%。经过多年努力，制约城市发展的东江收费站已被撤销拆除。市中心城区主干道改造、水电公园、江北东路（上任桥至九龙桥段）、河池西高速路口连接线、龙江河综合整治、龙江民俗街二期工程、老街桥改造等项目竣工投入使用，完成27条小街小巷改造。荣获“广西园林城市”和“南珠杯”竞赛特等奖。9个县（市）4个乡镇获得“南珠杯”竞赛奖励。11个镇入选全国重点镇。9个乡改为镇建制。扎实开展“美丽河池·清洁乡村”活动，大力推广“党领民办、群众自治”模式，全面启动生态乡村活动，打造清洁乡村升级版。

出台加快推进生态文明建设实施意见，被国家确定为生态功能红线落地试点市。龙滩岩滩水库生态环境保护试点项目扎实推进，河池市土壤污染综合防治示范区列入国家6个示范区之一，已全面启动建设。实施黄标车、老旧车淘汰工作，污水处理设施建设加快推进，有色金属、畜禽养殖等重点领域污染物减排工作不断加强，二氧化硫、氮氧化物减排完成全年任务。治理岩溶面积538平方公里，治理石漠化面积278平方公里。森林覆盖率增加0.24个百分点，达68.47%。全市环境质量持续向好，中心城区空气质量优良率达97.8%，PM10指标全区排名由2013年的第10位提升为第5位，主要河流断面水质达标率100%。

（七）保障和改善民生，群众生活水平不断提高。十项惠民工程全面完成。全市公共财政民生领域支出186.67亿元，占公共财政预算支出84%，科技、教育、卫生计生、文体、民政、社会保障等各项社会事业取得新成效。完成村（社区）“两委”换届选举。南丹、都安、罗城3县列入国家科技富民强县专项行动计划，每万人口发明专利拥有量增长57.3%。实施教育振兴行动计划，七大重点工程有序推进，基本解决中心城区义务教育“大班额”问题，九年义务教育巩固率达88.3%，市职业教育中心学校成功创建国家中等职业教育改革发展示范学校。南丹铜鼓舞勤泽格拉列入国家级非物质文化遗产代表性项目名录，10个项目列入自治区级名录。评选首次社会科学优秀成果52项，首届“刘三姐文学艺术奖”作品16件。罗城荣获全国文化先进县。巴马荣获全国计划生育优质服务先进单位。建设44处公共体育设施，全民健身活动实现常态化。全市城镇新增就业2.83万人，农村劳动力新增转移就业9.3万人次。企业退休人员养老金、城乡低保补助、市直事业单位离退休人员生活补贴、工伤职工伤残津贴等多项民生保障标准提高。社会保险参保人数115.8万人，基金征缴收入15.78亿元。新农合参合率99.46%。在全区率先实行农民工实名制登记和农民工工资保证金集中管理制度，切实维护农民工合法权益。全面完成保障性住房建设和农村危房改造任务。建立30个平价商店，充分发挥价格调节基金作用，物价保持总体稳定。投入1879万元治理78处地质灾害隐患，保障1万多名群众生命财产安全，地质灾害防治责任落实经验在全区推广。食品药品安全形势总体稳定，各项监管工作处于全区前列。在全区率先实行党政同责、一岗双责的安全生产责任制，安全生产形势持续向好。推广“天峨经验”，健全“一村一警”警务机制，实施中心城区“天网工程”改造，城区复合型警务机制改革和农村道路交通安全工作稳步推进。与毗邻接边地区建立联防联调机制，实现“四无”目标。社会大局和谐稳定，社会公众安全感连续15个季度在全区排名第二位以上。

（八）加强政府自身建设，行政效能持续提升。扎实开展党的群众路线教育实践活动，通过开展简政放权、惠民利民、清风正气、精减政务“四大行动”，解决一批基层、企业和群众反映强烈的问题，完善一批为民务实清廉的制度。完成市县两级政府机构改革，政府职能转变各项工作有序推进，初步形成权界清晰、分工合理、权责一致、运转高效、法治保障的政府职能体系。深化政务公开，网络问政形成常态。全面推进简政放权，取消、下放和调整合并市本级行政审批事项40项。在全区首创政务服务与公共资源交易一体化管理模式运行成效显著，市政务服务中心受理各类审批事项按时办结率100%，群众满意率99.9%，市公共资源交易中心组织完成交易项目329个，总成交额22.95亿元，节约资金1.42亿元。加强审计监管，财政资金使用效率提高。停止新建和改扩建政府性楼堂馆所。“三公”经费下降11.96%。全力推进清廉政府建设，群众对党风廉政建设和反腐败工作满意度提升1.3个百分点，达82.4%，高于全区3.7个百分点。认真执行市人大及其常委会的决议、决定，自觉接受人大及其常委会的法律监督和工作监督，自觉接受人民政协的民主监督，人大代表议案、建议和政协提案办结率100%。

一年来，民族事业全面进步，民族关系和谐稳定，成功举办罗城仫佬族自治县和9个民族乡成立30周年庆祝活动，4个集体和4名个人分别获得全国民族团结进步模范集体和模范个人荣誉称号。成功承办韦拔群同志诞辰120周年纪念活动。不断加强国防教育、国防后备力量建设，“国防动员委员会”在市人民政府挂牌，双拥共建工作进一步深化。统计、监察、编制、法制、司法行政、外事侨务、宗教、人防、机关事务、档案、地方志、防震减灾、粮食、供销等工作迈出新步伐，妇女儿童、老干部、老龄、残疾人保障等事业取得新进展，驻河池中直、区直各单位取得新成绩，多个单位、部门荣获全国或自治区级荣誉。

各位代表！回顾过去一年的工作，我们深切体会到，要保持我市经济社会持续健康发展，必须坚持牢牢把握发展第一要务，坚持主题主线，不断提高发展的质量和效益，着力推动转型跨越；必须坚持深化改革开放，破除制约科学发展的体制机制障碍，不断增强发展的动力和活力；必须坚持牢固树立保护生态环境就是保护生产力、改善生态环境就是发展生产力的理念，在保护中发展、在发展中保护；必须坚持一切为了人民群众、一切依靠人民群众，着力解决好人民群众最关心最直接最现实的利益问题，切实保障和改善民生；必须坚持统筹兼顾、突出重点，立足当前、着眼长远，紧紧抓住重大基础设施建设、生态环境保护、重点工程推进等事关全局和长远发展的大事，以重点工作带动整体工作，促进经济社会全面发展。

各位代表！成绩来之不易，这是市委正确领导的结果，是全市人民团结拼搏的结果，是社会各界共同努力的结果。在此，我谨代表市人民政府，向全市各族人民，向人大代表、政协委员，向各民主党派、工商联和社会各界人士，向驻河池中直、区直各单位和驻河池部队，向所有关心支持河池发展的朋友们，致以崇高的敬意和衷心的感谢！

在肯定成绩的同时，我们也清醒地看到，我市经济社会发展还存在不少突出矛盾和问题：一是受宏观环境和市场需求影响，经济下行压力加大，大项目支撑少，投资增长后劲不足，城乡居民增收渠道单一，进出口贸易大幅萎缩，虽然年初确定的各项约束性指标全面完成，但少数预期性指标与年初目标尚有差距。如，全社会固定资产投资低于预期目标2个百分点，城镇居民可支配收入低于预期目标0.5个百分点，农民人均纯收入低于预期目标2个百分点，外贸进出口总额低于预期目标16.4个百分点。二是产业结构不够合理，抗市场风险能力较弱，资源优势仍没有完全转化为经济优势。三是新的增长点不多，新兴产业仍处于培育阶段。四是贫困人口仍然较多，贫困面较大，贫困程度较深，开发扶贫任务艰巨。五是发展环境仍需改善，还存在少数干部不务实，个别干部不廉洁甚至腐败等问题。对于这些困难和问题，我们将以对人民高度负责的态度，以勇于担当的精神，积极采取有效措施，努力克服和解决，不辜负全市各族人民的期望！

二、2015年工作安排

今年是全面深化改革的关键之年，是全面推进河池法治建设的开局之年，也是全面完成“十二五”规划的收官之年。做好今年的工作，意义重大。

当前，经济发展进入新常态，发展的速度、方式、结构、动力、机制正发生重大转变。河池正面临新的重大机遇：左右江革命老区振兴规划即将出台，自治区实施“双核驱动、三区统筹”区域发展战略布局，中央、自治区对河池发展的关心支持力度更大。我市交通基础设施明显改善，地处西南出海大通道咽喉要塞的区位优势进一步凸显，丰富独特的资源优势进一步发挥，全市人民求发展、盼富裕、奔小康的愿望更加强烈。我们一定要把握区位优势，抢抓重大机遇，树立责任意识，乘势而上，实干争先，适应新常态，引领新常态，提高新常态下推动发展的能力和水平，奋力开创经济社会发展的崭新局面！

政府工作总体要求是：深入贯彻党的十八大、十八届三中、四中全会和习近平总书记系列重要讲话精神，以邓小平理论、“三个代表”重要思想、科学发展观为指导，紧紧围绕“建设幸福新河池”目标，坚持以提高经济发展质量和效益为中心，主动适应经济发展新常态，坚持稳进突围、提质发展“一个基调”，用好党的群众路线教育实践成果巩固年活动和“五大工程提升年”活动“两个抓手”，推进工业园区建设、开发扶贫创新、巴马长寿养生国际旅游区建设“三个突围”，营造抓落实重实干、抓建设促发展、抓责任敢担当、抓纪律树新风“四个风气”，促进经济、政治、文化、社会、生态文明建设“五个发展”。

经济社会发展主要预期目标是：地区生产总值增长7%，财政收入增长8%，规模以上工业增加值增长13%，固定资产投资增长15%，社会消费品零售总额增长11%，招商引资内资到位增长8%、外资到位增长20%，城镇居民人均可支配收入增长9%，农村居民人均纯收入增长10%，居民消费价格涨幅控制在3.5%以内，节能减排指标控制在自治区下达的目标范围内。

为确保实现今年的目标任务，必须深入开展党的群众路线教育实践成果巩固年和“五大工程提升年”活动，重点抓好以下工作：

（一）推进园区建设突围，加快工业转型升级。

以壮大总量、调整存量、做优增量为主攻方向，提升园区基础设施竞争力、环境竞争力、区位及资本集聚竞争力，加快新型工业化步伐。

继续集中力量加快园区建设。以大任产业园、都安临港工业园、南丹工业园、宜州经济开发区、环江工业园等5个市级工业园区为重点，大力推进园区基础设施和项目建设。其中，大任产业园入园二级公路竣工通车，4条园内路网贯通，供水工程、110千伏送变电站一期工程力争交付使用，华远、金兴力争试产，其他首批入园企业和污水处理设施主体工程基本完成。大力支持县级工业园区加快建设。启动天峨·罗甸桂黔红水河工贸园、南丹·独山民族工业园、环江·荔波民族生态产业园建设，打造黔桂（黔南、河池）石漠化连片特困地区合作发展示范区。全市工业园区（集中区）基础设施建设完成投资7亿元以上，工业总产值实现235亿元。

大力推进传统产业转型升级。继续实施《广西河池生态环保型有色金属产业示范基地规划》，加快发展锡基、锑基、锌基、铟靶材料等新材料产业，推进南方集团锑银多金属回收、南丹明鑫公司冶炼废渣综合利用、环江富鑫公司固废综合利用等一批有色金属深加工、环境治理和综合利用项目建设，整合提质有色金属产业，年产值实现140亿元以上。推进环江、罗城、天峨等风电项目，积极探索和推动直供电工作，支持鼓励企业利用余热发电，建设自备电厂，电力产业年产值力争保持100亿元。加快广维公司生物质制聚乙烯醇、河化集团技改等项目建设，延长生物化工产业链，年产值实现22亿元。支持宜州博庆公司“精制糖”和都安永鑫公司“黄金砂糖”扩能提产，多渠道整合糖蜜、蔗渣资源，促进广维、博冠等公司生产。延长茧丝绸产业链，加快构建桑蚕—茧丝—丝绸—印染—服装一体化丝绸工业新体系。推动碳酸钙产业发展，竣工投产西江鱼峰新型干法水泥一期工程。加快发展汽车零配件、汽车装饰品综合配套能力，大力发展新能源电动汽车。制糖、茧丝绸、建材、机械产业年产值分别实现35亿元、26亿元、17亿元、6亿元。

努力壮大特色和新兴产业。按照“一区多园”、“园中园”模式，以重点企业、重点产业带动园区发展。推进丹泉名酒文化城、中天领御酒业公司中天酒庄、天龙泉酒业公司技改扩建等项目建设，打造白、红、黄、清和保健并进发展、特色突出的南丹、罗城、宜州、都安、东兰等5个区域酒业中心和产业集群。酿酒产业年产值实现37亿元。重点推进九千万、巴马丽琅技改扩建，做大康之源高端饮用水等项目，整合提升优质饮用水产业，加快形成集群发展格局。优质饮用水产业年产值实现7亿元。依托绿色长寿生态资源优势，开发保健养生食品，提升医药产业规模和品牌影响力。长寿食品和医药产业年产值实现7亿元。积极培育发展新材料、新能源、电子信息等高新技术产业。

深入推进“抓大壮小扶微”工程。完善中小企业助保金管理办法，推进重点企业直通车服务制度，深化市领导联系重点企业机制，继续开展“市长企业接待日”活动，全面落实小微企业扶持政策，进一步优化发展环境。年内培育规模以上工业企业15家以上。

（二）强化现代农业示范，力促农业提质增效。

毫不松懈稳定粮食生产。严格保护基本农田，完成永久基本农田划定工作。大力开展粮食高产创建，推广粮食生产综合集成新技术，促进粮食生产增产增效。全市粮食种植面积稳定在410万亩，总产量100万吨以上。

抓好现代特色农业（核心）示范区建设。按照经营组织化、装备设施化、生产标准化、要素集成化、特色产业化“五化”要求，对通过自治区验收的现代特色农业（核心）示范区，重点完善生产经营组织机制，强化基础设施建设，提升示范区档次和科技含量，带动农业增效和农民增收，从核心示范区向拓展区发展。其他县（市、区）完善示范区建设规划，统筹发展特色产业、基础设施、休闲农业、村容村貌，加大推进示范区建设力度。核心示范区连片种植面积3000亩以上、拓展区5000亩以上、辐射区面积10000亩以上。核心示范区农民人均纯收入比拓展区高10%以上，比辐射区高15%以上，比所在乡镇高20%以上。力争各示范区建设均达到自治区验收标准，获得自治区认定。

大力发展特色优势产业。围绕构建“两带一区”农业发展布局，大力推行特早熟、特晚熟、特优质“三特”农业，促进错季生产、错峰上市、错位发展，做大做优特色产业。建设“双高”糖料蔗示范基地3万亩，糖料蔗种植面积稳定在100万亩；扩种桑园1.45万亩、总面积达80万亩；建设天峨珍珠李示范基地3万亩，新种特色水果2万亩、水果总面积80万亩；蔬菜种植面积120万亩，常年蔬菜基地6500亩以上；烤烟种植面积3.6万亩；食用菌种植规模1.5亿棒以上；做大香猪、菜牛、山羊等特色养殖业，出栏香猪120万头、菜牛20.5万头、山羊83万只；发展库区大水面养殖，大水面渔业产量3.2万吨。积极推进富硒农产品开发，打造以巴马为中心的富硒农产品基地。大力发展林下经济，产值实现38亿元以上。新增认证“三品一标”产品10个、面积26万亩。

夯实农业发展基础。加固7座病险水库，建设中小河流堤防42公里。新增和恢复灌溉面积4.2万亩，改善灌溉面积14.4万亩。解决33.7万农村人口饮水不安全问题。强化农机推广服务，农作物耕种收综合机械化水平达40%以上。大力推进农业科技进步，推广增产增效新技术和农业清洁生产技术，主要农作物优质良种覆盖率96%以上。

（三）加快发展以旅游业为重点的第三产业，提升服务业发展水平。

加快发展旅游业。不断完善巴马长寿养生国际旅游区规划体系，强力推进旅游区基础设施大会战项目建设，重点推进巴马国际旅游区集散中心项目一期工程、命河—水晶宫5A级景区基础设施、东兰坡豪湖国家湿地公园公共服务设施建设项目一期工程、凤山三门海景区寿源山庄、天峨大山原始森林景区旅游基础设施、都安澄江国家湿地公园、大化红水河百里画廊养生度假区等重大旅游项目建设，着力推动旅游区一体化发展。统筹推动“（南）丹金（城江）宜（州）罗（城）环（江）”板块旅游提质发展，以刘三姐文化旅游为引领，推动少数民族文化旅游业加快发展。巴马、宜州力争成功创建广西特色旅游名县。设立河池市拓展旅游市场基金，建立市级旅游宣传推广体系，加强旅游景区环境综合整治，打响“河池长寿地•三姐文化城”品牌。深入开展“比服务赛质量，争一流创十佳”活动，努力提升旅游服务质量，年内力争创建A级景区、星级饭店各2家以上。全年接待游客人数、旅游总收入分别增长15%、18%以上。

大力发展文化产业。落实文化产业发展行动计划，推进文化产业结构调整，力促文化与旅游、科技、体育等深度融合，加快发展创意文化、动漫游戏、数字出版等新兴文化产业。加快旅游纪念品和工艺品研发，重点发展铜鼓、刺绣、民族服饰、竹藤芒编、奇石等工艺品制造业，重点推进宜州、都安、罗城、东兰特色文化产业县（市）建设。文化体育固定资产投资完成2.3亿元。

推动商贸物流业发展。加快建设河池粮食物流产业园、河池城东百货家居城市综合体、市中心城区商品批发配送中心项目（汇金国际广场一期工程）、鸿宇建材城二期工程和东兰、南丹、都安等专业市场。开工建设宜州东盟国际茧丝绸交易中心。建成3个乡镇商贸中心和4个供销系统“新农村现代流通服务网络工程”项目，完善农村市场体系建设。引导和推动矿泉水、茧丝绸、农副产品、农业机械、有色金属新型材料深加工产品出口。培育新增自营进出口权企业2家。

提升金融业和信息服务业发展水平。积极“引金入河”，新增小额贷款公司、银行网点各1家。年内新增贷款40亿元以上。开展能繁母猪、水稻、糖料蔗、林业等政策性保险，扩大农业保险覆盖面。加快网络提速升级，新增20兆以上带宽覆盖住户4万户，新增宽带端口3万个。提高企业信息化应用水平，新增移动网络专线500条，为1000家中小企业提供有线或无线接入能力。加快推进乡村网络建设，实施乡镇所在地光网建设项目，提升行政村通宽带覆盖率。

积极扩大消费需求。采取内展销、外推介方式，组织开展节庆促消费活动。实施“电商广西”“电商东盟”工程，积极发展电子商务、连锁经营、物流配送等新型流通业态。拓展文化、体育健身、娱乐、旅游、信息等新兴服务消费。规范房地产市场秩序，推动物业管理市场提质扩面，促进房地产市场平稳健康发展，完成房地产开发建设投资25亿元。深入实施 “菜篮子”工程，确保全市生活必需品市场有效供应，物价稳定。坚决打击商业欺诈、制假售假行为，让广大群众方便消费、放心消费、安全消费。

（四）继续深化开发扶贫创新，加快全市脱贫步伐。

瞄准主要矛盾和关键问题，整合资源，加大投入，精准发力，推动开发扶贫创新突围。年内减少农村贫困人口16.5万人以上，力争全市农村贫困人口下降到65万人左右。

强力发展扶贫产业。围绕十大扶贫主导产业，组织实施广西“十百千”产业化扶贫示范工程项目，以产业发展为重点，稳步推进精准扶贫，确保每户贫困户至少有1项产业增收项目。因地制宜发展“短平快”项目，兼顾长效产业开发，进一步打牢群众增收基础。继续“整市推进”核桃产业，完成37万亩核桃种植任务，总面积突破200万亩，加强核桃管护和示范工作，提高成活率和挂果率，每个县（市、区）创建示范基地1万亩，乡（镇）创建示范基地1000亩，村创建示范基地100亩，种植户重点示范管护10株。

推进连片开发扶贫。抓好市级（大安）“整乡推进”示范区后续建设，各县（市、区）继续抓好各1个“整乡推进”示范点建设，示范点农民人均纯收入增幅高于当地平均水平。完成“十二五”时期436个贫困村“整村推进”开发扶贫工作任务，谋划“十三五”“整村推进”开发扶贫工作。深入实施乡村旅游开发扶贫“十百千”示范工程，新增星级农家乐、星级乡村旅游区各2家以上。完成《滇桂黔石漠化区域发展与扶贫攻坚河池片区实施规划（2011—2015年）》目标任务，治理岩溶380平方公里、治理石漠化150平方公里。全面完成岩滩库区移民遗留问题处理规划基础设施项目建设任务，完成投资7亿元。

推进生态移民。加快建设自治区层面统筹推进的大化、都安易地扶贫搬迁与城镇化结合试点工程，开工建设环江扶贫生态民族安置工程、天峨扶贫生态移民新城等项目，搬迁安置1754户8010人。

大力发展劳务经济。积极开展技能培训，提高农民科技致富能力和转移就业能力，全年开展农民科技培训20万人次，新型职业农民培育1000人次，职业学历教育培训2000人。推进农村劳动力转移就业实名制登记，继续集中管理农民工工资保证金，维护农民工合法权益，促进农村劳动力有序转移和稳定就业，全市农村劳动力新增转移就业6万人次以上。

（五）狠抓“十三五规划”和交通项目建设，夯实发展基础。

深化领导干部联系重大项目制度，继续实施重大项目并联审批、容缺预审、进展公示、绩效考评、督查通报、约谈警示等制度，推动重大项目加快建设。

研究制定“十三五”规划。认真总结“十二五”规划实施情况，精心编制“十三五”规划，努力争取我市重大工程、重大政策和重大项目得到国家和自治区更多支持。加大汇报对接力度，力争列入左右江革命老区重大工程建设攻坚3年行动计划的重大项目尽早开工建设。

继续掀起交通建设新高潮。继续坚持“抓好铁（路）公（路）机（场）、打通金水道”，加快推进一批重大交通项目建设。铁路方面：配合有关部门，力争开工建设贵阳经河池至南宁客运专线，推进黔桂铁路增建二线项目前期工作。公路方面：加快河池至百色高速公路建设，完成投资15亿元以上；配合开展南丹至下老、来宾至巴马、融水至河池等高速公路项目的前期工作。建成大化至巴马、罗城至柳城（河池段）二级公路；加快推进凤山至凌云（逻楼）、天峨至凤山（凤山段）、南丹（吾隘）至东兰、巴马至平果（坡造）、都安至武鸣、环江至东江、都安绕城线、三岔经洛东至宜州、东兰泗孟至武篆二级公路等14条重点路网项目和县乡联网公路项目。争取开工贵州瑶山至南丹、天峨至凤山（天峨段）、罗城经怀宝至融水洞头（罗城段）、东兰绕城公路等路网项目。修建通113个建制村约900公里通村沥青（水泥）路，除29个搬迁和新建的建制村外，其余建制村全部通沥青（水泥）路，修建595条957公里屯级路，85%以上自然屯（20户以上）通屯级路。实施一批危桥改造、安保工程、乡镇客运站、便民码头、城区客运站改造等项目。搬迁金城江客运服务总站。机场方面：增加河池机场国内航线，拓宽客源，提升质量。推进巴马、宜州2个通用机场项目前期工作。黄金水道建设方面：建成都安港区红渡作业区技改项目一期工程，开工南丹港吾隘作业区项目。配合推进龙滩升船机工程、龙滩坝上坝下码头工程，争取开工建设。

（六）推进新型城镇化建设，不断优化人居环境。

加强城乡规划修编。形成河池市城镇体系规划。调整完善河池市土地利用总体规划。完成河池市、宜州、罗城、东兰、巴马、都安城市总体规划修编正式成果报批。实现控制性详细规划对市中心城区和各县（市）城区建设用地范围的全覆盖。完成乡镇总体规划和详细规划的编制，实现详细规划对建制镇政府所在地的全覆盖，全面完成辖区内所有行政村及重点自然屯村庄规划设计。稳妥推进宜州新区规划和建设，推动宜州—金城江同城化发展。

加快推进市中心城区建设。竣工芝田、百旺等路网，加快河池碧桂园、工人文化宫、市中医医院住院综合楼、市老年大学、市体育场等一批项目建设，提速城东、城西片区发展。开工建设老虎山、人民厂转盘、铜厂转盘、南桥转盘等四大片区棚户区改造项目，提质旧城改造。完成金城中路（思源路口至南桥红绿灯段）、解放东路、解放西路（九龙桥至铁路抽水泵站段）、南新西路（联通转盘至文体路口）、江北西路至吉腰粮库等道路改造；贯通江南西路（水厂码头至老街桥段）、金福路（市中心血站至金城江区运管局段）、星河路（八小至市烟草局宿舍区段）；开工虎山路、翠竹路、江南东路（龙江桥至九龙桥段）、动物园路（金城江电池厂至河池供电局段）等道路项目，建设辉乐桥（六桥），尽快贯通影响群众出行的“断头路”，形成“一江两岸”循环路网，改善中心城区交通。完成南新东路（糖厂坡至金城江农贸市场段）、南新西路（人民厂转盘至南桥市场段）雨污分流改造建设，全力做好小街小巷硬化、亮化、美化改造，改善人居环境。

提质扩容县城和小城镇建设。按照规模适度、布局合理、突出特色、集约发展的原则，提质扩容发展县城和重点镇，推进以人为核心的新型城镇化，各县（市）重点打造1个示范新区。加快建设一批特色小镇、名村，重点推进宜州怀远镇、罗城龙岸镇、环江洛阳镇、南丹车河镇和吾隘镇同贡村、天峨向阳镇和六排镇云榜村、大化都阳镇、凤山三门海镇坡心村、东兰三石镇弄英村、巴马甲篆镇平安村达勒屯等11个自治区百镇示范工程和特色名村建设。完成城乡新貌新风工程项目投资42亿元以上。

提高城镇建设管理水平。持续推进市中心城区环境卫生、粉尘污染、交通综合、大气污染、户外广告、占道经营、饮食摊点排污等7个专项整治，建立长效机制，促使城市容貌整洁有序，争创“国家园林城市”。强化“两违”综合整治和城镇环境综合整治，加强对城市公共设施的管理和维护，坚持“门前三包”长效管理机制，规范建筑市场管理。

（七）全面加强环境保护，建设生态文明模范市。

深入开展“生态乡村”活动。巩固拓展“清洁乡村”活动成果，全面实施以村屯绿化、饮水净化、道路硬化为重点的“美丽河池·生态乡村”活动。加快宅旁、村旁、路旁、水旁等“四旁”绿化，完成450个绿化示范村屯建设任务，以点带面提升农村绿化整体水平。农村自来水普及率67%以上。完成500个村屯（50户以上）主干道硬化。

大力推进生态文明建设。配合自治区人大常委会审议通过《广西壮族自治区巴马盘阳河流域生态环境保护条例》并认真组织实施，保护好巴马国际旅游区的珍稀资源。开展生态环境调查和生态功能红线落地试点工作。划定市中心城区黄标车限行区域，市中心城区空气质量持续稳定达到二级标准。加强市县两级集中式饮用水水源保护。推进重金属污染防治、龙滩岩滩水库生态环境保护项目，加快国家级土壤污染综合防治示范区建设。深入实施“绿满八桂” 、“珠江防护林”等造林绿化工程，全市森林覆盖率68.6%以上。加强环境监管执法，坚决查处清理违规违法企业，实行环境监管工作网格化管理，不留盲点和空白区，坚决防范重特大环境污染事件发生。

强化节能减排工作。大力发展生态经济和循环经济，推广节能减排新技术、新产品、新工艺、新材料的应用。加快推进城镇污水和生活垃圾处理设施、企业污染减排、规模化畜禽养殖污染物治理项目建设。强化污染源在线监控，进一步控制污染物排放，全面完成“十二五”节能减排任务。

（八）深化改革扩大开放，激活发展强大动力。

广泛凝聚改革共识，牢牢把握改革方向，继续全力推进改革，全面扩大开放，着力增强发展的动力与活力。

以更有力的举措深化改革。按照中央和自治区的统一部署，紧密结合我市实际，抓紧推进以下几个重点领域的改革：深化行政审批制度改革。加快政府职能转变，充分发挥市场在资源配置中的决定性作用，更好地发挥政府公共服务、市场监管、社会治理、环境保护的作用。进一步简政放权，提高政府行政效率。加强政务服务体系和政务服务中心基础设施建设，建立健全全市统一的电子政务平台，受理审批事项按时办结率达100%。稳步推进政府购买服务。深化农村改革。推进11个乡（镇）土地确权登记颁证试点工作并全面铺开，试点乡镇农户发证率80%以上。加强农村土地承包合同管理，积极稳妥有序推进以“小块并大块”为重点的土地集中流转。继续深化集体林权制度改革。培育壮大新型农业生产经营组织。加大对农村专业大户、家庭农场、农民合作组织的扶持力度，鼓励和支持承包土地向农业新型经营主体流转，发展适度规模经营。培育年销售收入5000万元以上的农业产业化龙头企业45家以上，新增农民合作社150家，新增家庭农场50家。深化土地管理制度改革。加快推进农村地籍调查和集体建设用地使用权确权登记发证工作，开展不动产统一登记工作。加快推进征地制度改革，完善对被征地农民合理、规范、多元保障机制，建立和完善兼顾国家、集体和个人的土地增值收益的征地制度。深化国资国企改革。积极发展混合所有制经济，推动经营性国有资产集中统一监管，全面实现政企分开、政资分开和事企分开。按照现代企业制度要求，健全完善公司法人治理结构，进一步落实外派监事会制度。以选聘制、任期制为主要方式，深化国有企业领导人员管理体制改革，加强县（市、区）国资监管，做大做强国有经济。依法稳妥处置国企改革遗留问题。深化工业园区体制机制改革。探索园区市场化运作、企业化管理模式，进一步完善园区管理体制，支持园区按照法律法规要求在管辖区域内进行自主创新试点。突出市场主体地位，发挥政府统筹作用，理顺园区与各方面的关系，提升园区自主发展能力。试行项目建设全程协办、代办机制，全面提高行政效率、提升服务质量，推动园区建设突围和项目落地。深化金融体制改革。推进农村金融改革，宜州、南丹、大化3县（市）创建“农村金融创新示范县”，开展“信用巴马”社会信用体系创建工作。加大信贷金融扶贫合作与创新力度，实施扶贫小额贷款奖补和贫困村互助金试点，建立金融服务“三农”和小微企业长效机制。深化城镇化发展体制机制改革。进一步放宽城镇户籍限制，鼓励农民工就近有序转移。创建多元可持续的城镇建设资金投入机制，发行第二期14亿元城投企业债券，吸引更多社会资本参与城市公用设施投资、建设、运营。同时，统筹推进文化、教育、卫生、司法等领域改革。开展公务用车改革工作。

以更开阔的视野扩大开放。加快发展开放型经济，优化开放格局，积极融入“双核驱动、三区统筹”战略布局，继续实施“柳来河一体化”规划，以开放促改革、促发展、促转型。推进中国—东盟博览会和各类招商引资签约活动重点签约项目的落地服务工作，项目履约率90%以上。加大招商引资力度，年内新签约项目金额200亿元以上，完成招商引资内资到位269亿元，外资到位1.2亿美元。

以更高度的自觉推动创新。实施创新驱动发展战略，以科技创新促进产业转型升级，建成1家科技企业孵化器，实施科技创新项目10个，新增自治区级企业技术中心1家、研发中心1家。鼓励和支持企业加大新产品、新工艺研发力度，提高企业自主创新能力，争创更多河池品牌。加强知识产权保护，营造鼓励创新、保护创新的良好氛围。

以更优质的服务发展非公经济。认真兑现和落实支持非公经济加快发展的各项优惠政策，继续用好“河池非公企业之声”、“银行+商会+企业”融资合作、信息发布、对外联谊交流等4个非公企业服务平台，引导扶持非公企业做大做强。

（九）着力保障和改善民生，提高群众幸福指数。

优先发展教育。继续深入推进教育振兴行动计划七大重点工程建设，九年义务教育巩固率、高中阶段毛入学率、学前三年毛入园率分别实现88.5%、80%、65%。鼓励民办教育，加快发展高等教育，关心支持特殊教育。加强教师队伍建设，培训各级各类教师2.6万名。解决好进城务工人员子女接受义务教育问题，关爱农村留守儿童，重视校园安全工作。

进一步扩大就业。继续实施农民收入倍增计划和城镇居民收入倍增计划，推动城乡居民特别是中低收入群体收入较快增长。认真落实鼓励支持就业创业的各项政策，重点抓好高校毕业生、返乡农民工等群体的就业创业工作。全市城镇新增就业1.8万人，城镇登记失业率控制在4.5%以内。

健全社会保障体系。推进社会保险扩面征缴，全市社会保险参保人数108.95万人以上。建立覆盖全市的 “五险合一”社会保险经办监管体系。大力推进新型农村社会养老保险和城镇居民社会养老保险制度合并实施工作。城乡居民基本养老保险参保率95%，缴费率90%，养老金发放率100%。继续推进河池市老年公寓综合楼、农村养老服务中心建设，建设65个农村幸福院、82个村级示范性老年协会。建成金城江区、天峨县救灾物资仓库，创建防灾减灾示范社区。落实重点优抚对象抚恤补助标准自然增长机制，全面实施优抚对象医疗救助一站式结算。积极发展社会福利和慈善事业，进一步做好残联、红十字会工作。

加快发展文化体育事业。大力弘扬中华优秀传统文化，牢固树立社会主义核心价值观。加强城乡文化、广播、体育基础设施建设。加强重点文物和非物质文化遗产保护。充分发挥民间文艺团体作用，丰富群众精神文化生活。规范文化市场秩序，加强“扫黄打非”工作。广泛开展全民健身运动，促进群众体育和竞技体育全面发展。

抓好卫生计生工作。扎实开展基本公共卫生服务。提速新农合信息化管理平台建设，巩固全市新农合“一卡通”，参合率稳固在96%以上。全面推进县级公立医院综合改革，深入实施防艾攻坚工程，组织实施壮瑶医药振兴计划。深化诚信计生“六化”模式，综合治理出生人口性别比偏高问题。

继续集中财力办十件惠民实事、好事。1.教育惠民。1.3万名幼儿享受学前教育资助，19万名农村义务教育阶段家庭困难寄宿生补助生活费，4万名高中阶段学生免学费，资助2.5万名贫困生、0.8万名贫困大学新生，资助工作实现从学前教育到高等教育全覆盖。2.卫生惠民。人均公共卫生服务经费提高到35元/年，城乡居民电子健康档案建档率86%以上，儿童一类疫苗报告接种率95%以上，免费婚检率95%以上。3.社会保障惠民。巩固提高城乡低保补助标准，全市城乡低保对象月人均补差水平分别达260元和110元以上。实施残疾人托养“阳光家园计划”补助项目。提高城镇职工大病医疗统筹金最高支付限额、城镇居民门诊大病种类最高支付限额及住院医疗费用统筹金支付比例。4.安居惠民。新建城镇保障性住房11676套，分配入住5570户，新增发放租赁补贴365户，完成农村危房改造25000户。5.农网改造惠民。新建及改造农村电网35千伏线路67.5公里、10千伏线路375公里，确保95%以上农户用上电。6.土地整治惠民。改造坡耕地1万亩，综合治理水土流失面积80万亩，实施“旱改水”工程4500亩，建成高标准基本农田4万亩，完成中低产田地改良面积13万亩。7.农补惠民。全面落实农作物良种补贴、退耕还林补助、森林生态效益补偿、大中型水库移民后期扶持补助、林业和渔业用油补贴、能繁母猪等保险保费补贴、农机具购置补贴等各项惠农补贴，强农惠农资金全额发放到群众手中。8.生态惠民。完成植树造林50万亩，全民义务植树800万株，新建沼气池2170座。9.文体惠民。新建150个村级公共服务中心、30个村村通无线发射台站和73处公共体育基础设施。继续实施图书馆、文化馆（群众艺术馆）、博物馆（纪念馆）和乡镇文化站等公共文化服务基础设施免费开放服务工作。10.计生惠民。国家免费孕前优生健康检查目标人群参检率86%以上，村级“幸福家园”覆盖率100%，计划生育家庭办理爱心保险2.7万户以上。

（十）推动社会治理创新，确保社会和谐稳定。

全面推进河池法治建设。贯彻落实党的十八届四中全会和自治区党委十届五次全会、市委三届六次全会精神，依法维护人民权益、社会公平正义、民族团结和社会稳定大局，为加快建设幸福新河池提供有力法治保障。加强和改进政府法制工作，支持人民法院、检察院依法独立公正行使职权。建成市县乡村四级公共法律服务平台，做好法律服务和法律援助工作。扎实推进全民守法，坚持法治与德治相结合，创建具有河池特点的法治文化，促进全市各族人民学法尊法信法守法用法。

深化平安创建活动。强化重力治乱保民安理念，推广农村社会治安防控“天峨经验”，不断健全“一村一警”警务机制，着力抓好社会治安整治工作，积极推动社会治理创新，建立健全“网格化巡逻防控、智能化视频监控、社会化内部管控、一体化严防严控”的社会治安防控体系，加大矛盾纠纷排查化解力度，坚决打击各类刑事犯罪，推进新一轮禁毒人民战争，提升人民群众的安全感和满意度。进一步加强和完善应急管理体制机制建设，不断提高突发公共事件处置能力。继续完善网络问政、电话热线等接访方式，依法规范信访秩序，畅通信访渠道，健全就地解决群众合理诉求机制。抓好非公有制经济组织、社会组织的建设管理。加强流动人口和特殊人群服务管理。推进基层民主和基层政权建设。贯彻落实党的宗教政策，加强宗教事务管理。

加强安全生产和食品药品监管。进一步强化安全生产“红线”意识，全面落实“党政同责、一岗双责”制度和企业主体责任，突出抓好矿山、道路交通、危险化学品等重点行业领域的隐患排查治理，强化“打非治违”专项行动，加强安全生产应急救援能力建设，有效防范重特大安全事故。完善乡镇食品药品监管所、农村食品药品协管员、信息员工作机制，筑牢基层监管网底。加强食品药品日常监管，深入开展专项整治，加大打击食品药品违法犯罪行为，确保群众饮食用药安全。

发展民族团结进步事业。认真贯彻落实党和国家的民族政策，继续推进民族团结进步模范市建设，扶持人口较少民族加快发展。支持办好都安瑶族自治县成立60周年庆祝活动。

支持部队和国防建设工作。大力推进双拥共建活动，巩固和发展军政军民团结，努力争创全国双拥模范城和全区双拥模范城。

三、依法全面履行政府职能，建设法治政府和服务型政府

全面完成今年的各项任务，对政府工作提出了新的更高的要求。我们将进一步加强政府自身改革和建设，持续深入改进作风，营造抓落实重实干、抓建设促发展、抓责任敢担当、抓纪律树新风等“四种风气”，努力建设人民满意的法治政府和服务型政府。

严格依法行政，在建设法治政府上迈出新步伐。坚持在党的领导下、在法治轨道上开展工作，创新执法体制，完善执法程序，推进综合执法，严格执法责任，建立权责统一、权威高效的依法行政体制，推行政府法律顾问制度。自觉接受人大及其常委会的法律监督和工作监督，主动接受政协的民主监督。广泛听取各民主党派、工商联、无党派人士的意见和建议。打造职能科学、权责法定、执法严明、公开公正、廉洁高效、守法诚信的法治政府。

巩固教育实践成果，在建设服务型政府上树立新形象。开展党的群众路线教育实践成果巩固年活动，强化落实已制定的一批制度，深化“红卡惠民”机制，密切与人民群众的联系，维护人民群众切身利益。始终把群众满意作为第一标准，把更多精力放到服务发展上，把更多财力用到民生改善上，把更多的改革红利落到群众身上，着力打造群众满意的服务型政府。

强化权力制约监督，在建设清廉政府上展示新风气。严格遵守八项规定、党政机关厉行节约反对浪费条例等各项政策规定，严格“三公”经费支出，坚持艰苦奋斗，勤俭办一切事业。深化党政领导干部和国有企业领导人员经济责任审计，自觉履行“一岗双责”，加强对领导干部、政府机关、公共资源交易等重点领域和关键环节的监督，深化党政领导廉洁履职社会监督工作，推行政府部门权力清单制度，扎实推进惩治和预防腐败体系建设，打造清廉政府。

各位代表！今年全市经济社会发展任务繁重，使命光荣，责任重大。让我们在市委的坚强领导下，锐意进取，励精图治，奋发图强，为加快富民强市新跨越，建设幸福新河池而努力奋斗!
